# Issue Information

**DOI:** 10.1002/mbo3.13

**Published:** 2013-10-08

## Aims and Scope

*MicrobiologyOpen* is a peer-reviewed journal delivering rapid decisions and fast publication of microbial science, a field which is undergoing a profound and exciting evolution in this post-genomic era. The journal gives priority to reports of quality research, pure or applied, that further our understanding of microbial interactions and microbial processes. *MicrobiologyOpen* gives prompt and equal consideration to papers reporting theoretical, experimental, applied and descriptive work in all aspects of microbiology.

The journal aims to serve there search community by providing a vehicle for authors wishing to publish interesting and high quality work in both fundamental and applied microbiology. MicrobiologyOpen will consider submissions across eukaryotic microorganisms, prokaryotes (bacteria and archaea) and viruses infecting or interacting with microorganisms, including genetic, biochemical, biophysical, bioinformatic and structural analyses.

The journal features original research articles, reviews and editorials. Original research papers must report well-conducted research with conclusions supported by the data presented in the paper.

*MicrobiologyOpen* publishes papers submitted directly to the journal and those referred from a select group of prestigious journals published by Wiley-Blackwell.

*MicrobiologyOpen* is a Wiley Open Access journal, one of a new series of peer-reviewed titles publishing quality research with speed and efficiency. For further information visit the Wiley Open Access website at http://www.wileyopenaccess.com.

## Open Access and Copyright

All articles published by *MicrobiologyOpen* are fully open access: immediately freely available to read, download and share. All articles accepted from 14 August 2012 are published under the terms of the Creative Commons Attribution License. All articles accepted before this date were published under a Creative Commons Attribution Non-Commercial License. The Creative Commons Attribution License permits use, distribution and reproduction in any medium, provided the original work is properly cited and allows the commercial use of published articles. Copyright on any research article in a journal published by *MicrobiologyOpen* is retained by the author(s). Authors grant Wiley a license to publish the article and identify itself as the original publisher. Authors also grant any third party the right to use the article freely as long as its integrity is maintained and its original authors, citation details and publisher are identifi ed. Further information about open access license and copyright can be found at http://www.wileyopenaccess.com/details/content/12f25db4c87/Copyright–License.html.

## Purchasing Print Reprints

Print reprints of Wiley Open Access articles can be purchased from corporatesales@wiley.com.

## Disclaimer

The Publisher and Editors cannot be held responsible for errors or any consequences arising from the use of information contained in this journal; the views and opinions expressed do not necessarily refl ect those of the Publisher and Editors, neither does the publication of advertisements constitute any endorsements by the Publisher and Editors of the products advertised. Wiley Open Access articles posted to repositories or websites are without warranty from Wiley of any kind, either express or implied, including, but not limited to, warranties of merchantability, fitness for a particular purpose, or non-infringement. To the fullest extent permitted by law Wiley disclaims all liability for any loss or damage arising out of, or in connection with, the use of or inability to use the content.

